# Highly homogeneous zero-index metamaterials make devices more compact and perform better

**DOI:** 10.1038/s41377-024-01458-6

**Published:** 2024-05-06

**Authors:** Wei-Xiang Jiang

**Affiliations:** State Key Laboratory of Millimeter Waves, School of Information Science and Engineering, Southeast University, 210096 Nanjing, China

**Keywords:** Metamaterials, Photonic crystals

## Abstract

A highly homogeneous microwave zero-index metamaterial based on high-permittivity SrTiO_3_ ceramics is demonstrated to realize the small-aperture high-directivity antenna. Such a novel technique is a remarkable step forward to develop compact devices with better performance.

Metamaterials are artificial composites made by periodically or non-periodically arranging sub-wavelength unit cell structures, and have demonstrated exotic effective medium parameters that are impossible or hard to obtain in nature, such as negative permittivity^1^, negative permeability^2^, near-zero refractive index^3,4^, etc. Among the various, zero-index metamaterials (ZIMs) are of particular interest for their ability to homogenize the phase distribution of EM waves propagating through them. Benefit from such exciting property, ZIMs have been widely used for various applications, such as EM tunneling^5^, bending waveguide^6^, geometry-independent antenna^7^, perfect absorber^8^, free-space cloak^9^, and surface-emitting laser^10^.

ZIMs can be realized through near-zero permittivity (ENZ), near-zero permeability (MNZ), and near-zero permittivity and permeability (EMNZ), where EMNZ stands out due to the finite and designable impedance and therefore allows for better impedance matching with conventional media^11^. Typically, EMNZ behavior can be developed through classic fishnet metamaterials or Dirac-like cone-based ZIMs (DCZIMs). The fishnet metamaterials contain periodic metallic wires and resonators arranged in the form of fishnets, while the DCZIMs consist of a planar dielectric cylinders array. Compared to fishnet ZIMs, DCZIMs are not plagued by ohmic losses due to their all-dielectric geometry. However, due to low permittivity of used dielectric materials, it is challenging for both methods to satisfy the homogenization criteria of metamaterials^12^. This limitation greatly hinders the development of ZIM-based devices towards compactness, thinness, and better performance.

In a recently published paper in *eLight*^13^, a collaborative team led by Prof. Yang Li, Prof. Yue Li, Prof. Jingbo Sun, and Prof. Ji Zhou from Tsinghua University, along with Prof. Ming Bai from Beihang University proposed a new approach for designing and realizing the highly homogeneous microwave zero-index metamaterial (ZIM), in which the lattice constant *a* is only one-tenth of the free-space wavelength *λ*_0_. Compared to the traditional photonic crystal-based ZIMs with lattice constant of ~*λ*_0_/3, the proposed ZIM’s homogenization level is highly improved. This achievement was made by filling high-permittivity SrTiO_3_ ceramics with a microwave relative permittivity *ε*_r_ of over 290 in BaTiO_3_ (*ε*_r_ ≈ 25) background matrix. Based on the highly homogeneous ZIM, the small-aperture high-directivity antenna was designed and realized.

Figure 1 presents a comparative illustration of the conventional ZIM and the highly homogeneous ZIM. In general, the microwave DCZIMs are made by Al_2_O_3_ (*ε*_r_ = 10) pillars in an air matrix^14^, which results in the value of *a*/*λ*_0_ ranging from 0.3 to 0.5, and thus significantly restricts the ZIM’s homogenization level (Fig. 1a). Such a large *a*/*λ*_0_ limits the minimum surface area of the DCZIM to 1.5*λ*_0_ × 1.5*λ*_0_ because it needs a surface area larger than 3*a*×3*a* to behave as a bulk zero-index medium. To reduce lattice constant and improve uniformity, the authors have synthesized and characterized high-permittivity SrTiO_3_ ceramics. Based on the solid-state method with binder, the fabricated SrTiO_3_ ceramic showing a nearly dispersiveless *ε*_r_ from 292 to 294 and a loss tangent from 9 × 10^–4^ to 1.45 × 10^–3^ at frequencies ranging from 5 to 8 GHz. By using the high-permittivity SrTiO_3_ as an inclusion and BaTiO_3_ as the background matrix, a highly homogeneous DCZIM in the microwave regime was realized, as shown in Fig. 1b. Because the high-permittivity feature increasing effectively the degenerate wavelengths of the electric monopole mode and the magnetic dipole mode, the DCZIM shows a homogenization level as high as *a*/*λ*_0_ ≈ 0.1. Both the simulated and measured near fields indicate that the phase of the wave propagating through the DCZIM exhibits an almost perfect uniform distribution. Leveraging these advantages, the authors developed a high-directivity antenna by incorporating the highly homogeneous ZIM into a metallic waveguide, and results show that this antenna can achieve a directivity as high as 11.2 dB with an aperture size of 1.2*λ*_0_ × 1.2*λ*_0_.

**Fig. 1:**
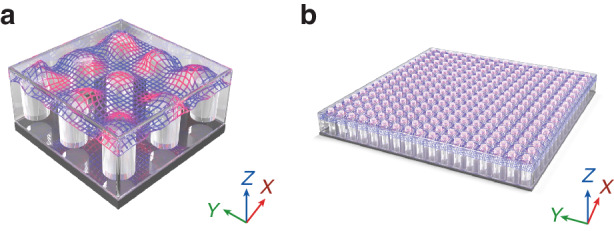
Schematic of zero-index materials. **a** Conventional DCZIM needs a surface area larger than 3*a*×3*a* to behave as a bulk zero-index medium. **b** Highly homogeneous DCZIM whose inclusion and background matrix permittivities are 294 and 25, respectively

The work presents an effective technology to implement the ZIMs with high homogenization and small size, which can improve performance and reduce feature size of the metamaterial-enabled devices, such as the aperture of antennas, the compactness of waveguides with arbitrary shapes, and the thickness of cloaks. In addition, the implementation and composite method of the high-permittivity materials could provide a reference for miniaturization of other advanced metamaterial technologies, such as programmable metasurfaces with real-time wave controls^15–17^, which can lead to various applications in wireless communications, imaging, and sensing.
